# Shunt Surgery for Pediatric Prehepatic Portal Hypertension: A Single-Center Case Series

**DOI:** 10.3390/jcm14217780

**Published:** 2025-11-02

**Authors:** Gabija Pikturnaitė, Austėja Račytė, Alina Rudokaitė, Gediminas Vaitėnas, Jonas Povilavičius, Benas Prušinskas, Ilona Dockienė, Marius Kurminas, Rūta Bernatavičienė, Gilvydas Verkauskas

**Affiliations:** 1 Faculty of Medicine, Vilnius University, 01513 Vilnius, Lithuania; austeja.racyte@mf.stud.vu.lt; 2 Children’s Surgery, Orthopaedic and Traumatology Centre, Vilnius University Hospital Santaros Klinikos, 08406 Vilnius, Lithuania; alina.rudokaite@santa.lt (A.R.); benas.prusinskas@santa.lt (B.P.); 3 Clinic of Cardiovascular Diseases, Faculty of Medicine, Vilnius University, 01513 Vilnius, Lithuania; gediminas.vaitenas@santa.lt; 4 Clinic of Gastroenterology, Nephro-Urology and Surgery, Faculty of Medicine, Vilnius University, 01513 Vilnius, Lithuania; jonas.povilavicius@santa.lt (J.P.); ruta.maciulyte@santa.lt (R.B.); gilvydas.verkauskas@santa.lt (G.V.); 5 Clinic of Anaesthesiology and Intensive Care, Faculty of Medicine, Vilnius University, 01513 Vilnius, Lithuania; ilona.dockiene@santa.lt; 6 Department of Radiology, Nuclear Medicine and Medical Physics, Faculty of Medicine, Vilnius University, 01513 Vilnius, Lithuania; marius.kurminas@santa.lt

**Keywords:** prehepatic portal hypertension, surgical shunts, Meso-Rex bypass, variceal bleeding, long-term results

## Abstract

**Background/Objectives**: Management of prehepatic portal hypertension involves endoscopic and medical therapies with subsequent shunting if symptoms persist. Lately, surgical shunts, particularly the Meso-Rex shunt, are increasingly considered early in the disease course, offering benefits such as minimized hyperammonemia, improved somatic growth, and preservation of liver function. Our study evaluates post-operative outcomes after different surgical procedures in children with prehepatic portal hypertension. **Methods**: This single-centre retrospective case series included six children undergoing surgical shunting for prehepatic portal hypertension over a 5-year period. Medical records before and after surgery, followed for an average of 4.0 years, were analyzed. **Results**: Five patients underwent a Meso-Rex bypass, while one patient underwent a mesorenal shunt procedure. All cases showed clinically significant regression of esophageal varices six months post-surgery. Thrombocyte as well as leukocyte count significantly increased in five out of six patients during the long-term follow-up. Currently, five out of six surgically formed shunts (83%) continue to function normally. **Conclusions**: Our study supports early surgical intervention for improved long-term outcomes in managing portal hypertension, reducing complications like hypersplenism and variceal bleeding. Early consideration and ongoing monitoring are crucial for long-term success in children with portal hypertension.

## 1. Introduction

Pediatric portal hypertension (PPH) is a rare syndrome defined as an abnormal increase in pressure in the portal vein system, usually caused by extrahepatic portal vein obstruction [1]. Although rare, PPH can lead to life-threatening complications, such as major bleeding from varices, and carries high mortality and morbidity [2,3].

Due to its low incidence, there is a lack of clinical trials evaluating different treatment strategies. To date, suggested primary management methods are endoscopic approaches and medical therapy to reduce portal pressure [4]. Surgical shunts are mainly recommended when medications or minimally invasive methods do not result in adequate improvement [5,6]. However, recent studies advocate surgical management early in the course of the disease, creating a new potential treatment paradigm for prehepatic portal hypertension with the prospect of reversing liver dysfunction [3,7,8,9].

The Meso-Rex shunt (MRS) is the only surgical shunt that restores physiological portal venous blood flow to the liver. In contrast to other shunts for PPH, MRS minimizes hyperammonemia and therefore does not adversely impact neurocognitive development after surgery. In addition, it reduces hypersplenism and improves previously impaired somatic growth [10]. However, in order to successfully perform an MRS, patients must have favourable vascular anatomy and an appropriate medical condition [8,11].

In this study, we present our experience with children undergoing shunting procedures for prehepatic portal hypertension at our tertiary care centre over a 5-year period. The aim of this case series was to evaluate post-operative outcomes after different surgical procedures in children with prehepatic portal hypertension.

## 2. Materials and Methods

This is a single-centre, retrospective analysis of post-operative outcomes of children who underwent surgical shunting for prehepatic portal hypertension over a 5-year period. The Regional Biomedical Research Ethics Committee approved this study on the 4 July 2023 (registration number 2023/7-1528-988).

In this study, pre-operative and post-operative medical records of 6 patients were analyzed. All patients needed to meet the following inclusion criteria: diagnosis of prehepatic portal hypertension and an elective shunting procedure performed according to standard care protocols. In our series, pre-hepatic portal hypertension was defined as portal hypertension caused by extrahepatic portal vein obstruction, in the absence of intrinsic liver disease. Practically, the diagnosis was established by a combination of clinical findings and imaging results. All patients had clinical signs of portal hypertension—such as splenomegaly with hypersplenism (thrombocytopenia/leukopenia) or variceal hemorrhage—and imaging evidence of portal vein blockage (portal vein thrombosis with cavernous transformation on ultrasound or occlusion on angiography). Liver function was normal in all cases, and there were no radiological signs of cirrhosis, confirming a pre-hepatic (extrahepatic) etiology. The indication for surgery in all cases was clinically significant hypersplenism and a persistent risk of gastrointestinal bleeding from esophageal varices despite prior medical and endoscopic management. Before surgical treatment, patients underwent endoscopic variceal sclerotherapy or band ligation as needed.

Prior to shunt implantation, all patients received computed tomography angiography to assess the Rex recess (intrahepatic left portal vein) patency. Vein portography was performed in a subset of patients when feasible to further evaluate portal anatomy.

After surgery, patients were followed for an average of 4.0 years. Follow-up visits were scheduled at 6 months post-surgery and at 1, 3, 4, and 5 years thereafter. Medical records were reviewed for demographic characteristics, risk factors, presenting symptoms, and type of shunt procedure. Pre- and post-operative laboratory values (platelet count, white blood cell count (WBC), aspartate aminotransferase (AST), and alanine aminotransferase (ALT)) were collected to assess changes in hypersplenism and liver function. Serial abdominal ultrasounds were conducted to track spleen size before and after surgery. Doppler ultrasonography was used to monitor shunt patency, with a postoperative shunt flow velocity expected to be at least 15 cm/s [9]. Shunt blood flow velocities up to ~40 cm/s were considered normal; significantly higher velocities could suggest shunt stenosis and prompted further evaluation with CT/MRI angiography [9,12,13]. Endoscopy was performed to evaluate esophageal varices preoperatively and during long-term follow-up.

Descriptive statistics were calculated and stored using Microsoft Excel for Mac, version 15.31 (Microsoft Corporation, Redmond, WA, USA). Long-term shunt patency, absence of further variceal bleeding, and improvement in laboratory test results were considered favourable postoperative outcomes.

## 3. Results

In total, six surgical procedures were performed at our surgical centre for children with prehepatic portal hypertension since 2018. A total of 50% (3) of the patients were male, and the mean age at surgery was 9.2 (SD ± 3.93) years. Demographic and medical characteristics for each case are shown in Table 1. Two patients were diagnosed with prehepatic portal hypertension incidentally (asymptomatic splenomegaly with thrombocytopenia). Three (50%) patients presented with hypersplenism symptoms (left upper abdominal pain, easy bruising, frequent infections). All patients developed varying degrees of esophageal varices over time, and four (67%) patients experienced episodes of variceal bleeding. Among these, variceal bleeding in three patients (patients 2, 3, and 6) was managed by multiple endoscopic sclerotherapy procedures, while one patient (patient 1) underwent a partial splenectomy followed by two additional band ligation procedures.

The average time from the diagnosis of prehepatic portal hypertension to surgical bypass interventions was 4 years (range: 2–9). All patients were ASA II–III and underwent general anesthesia, with regional techniques added in two cases (caudal anesthesia for a 4-year-old and spinal analgesia for a 16-year-old). The mean anesthesia time was 287 min, with slight blood loss (50–200 mL). Extubation was performed in the operating room, and all patients were admitted to the ICU for an average of 3 days (1–4 days). Postoperatively, one patient developed mild anemia, and two experienced transient nausea.

Five (83%) patients underwent a Meso-Rex bypass, and one (17%) patient underwent a mesorenal shunt procedure due to unfavourable anatomy for the Meso-Rex technique (left portal vein diameter < 2 mm). In four cases, the left internal jugular vein served as an interposed graft, while in one patient, the femoral vein was used, and in another, the gastric vein was used as a graft. Immediate postoperative Doppler assessment confirmed sufficient shunt blood flow in all patients. One patient (patient 4) with a gastric vein graft developed early shunt stenosis, requiring a relaparotomy and shunt dilatation three months after its initial placement.

All patients were followed, with an average follow-up period of 4.0 years. All cases showed clinically significant regression of esophageal varices by six months post-surgery. By the last follow-up, four (67%) out of six patients had no remaining esophageal varices (Table 2).

Throughout the first year after surgery, the patient with a Meso-Rex shunt using a gastric vein graft (patient 4) had persistent thrombocytopenia and splenomegaly despite adequate shunt flow (38 cm/s). This led to an additional intervention: partial splenic embolization. Subsequent follow-up showed improved blood counts in this case, with no further interventions required. Another patient (patient 1) was lost to follow-up after the initial post-surgical visit and returned 2.5 years later with a reduced platelet count and a slightly enlarged spleen compared to previous results, leading to consideration of partial splenic embolization as well. At the last visit, his shunt flow velocity was 13.5 cm/s, which is considered suboptimal. Postoperative shunt flow velocity for all patients, measured using Doppler ultrasonography at each visit, is presented in Table 3. Thus, currently, five out of six surgically formed shunts (83%) continue to function normally (Table 4).

**Figure 1:**
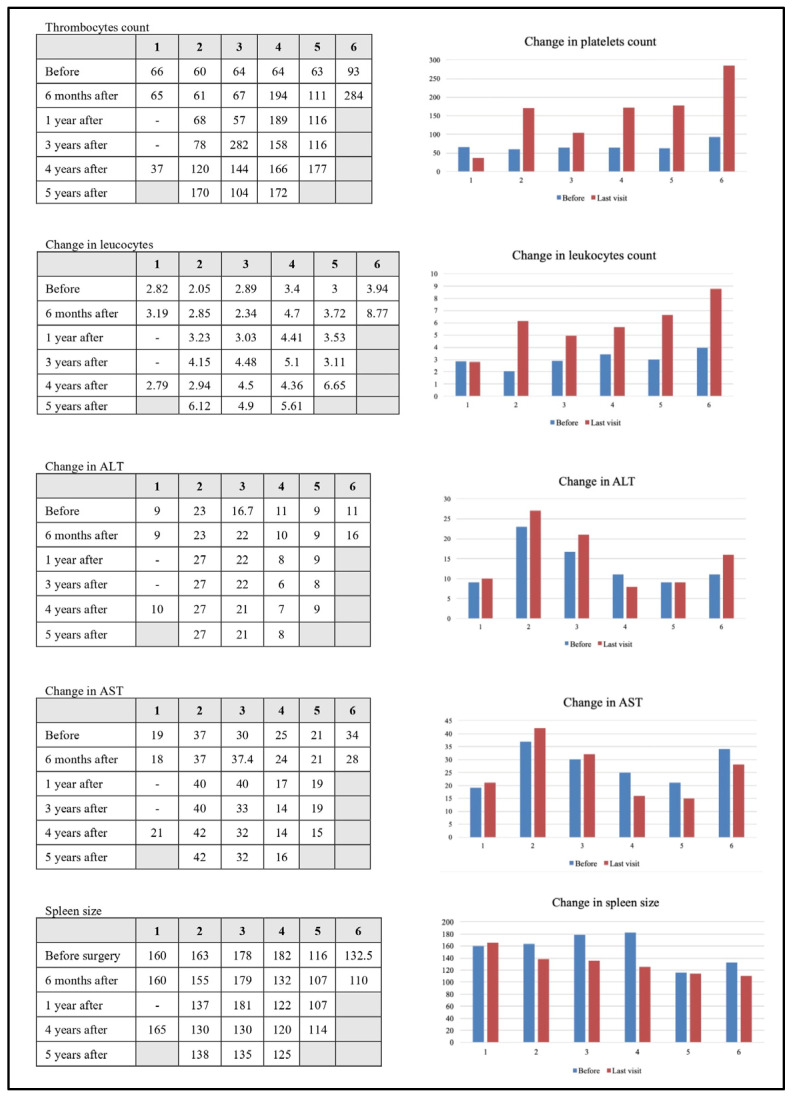
Detailed laboratory results according to visits.

Pre-operative and post-operative laboratory results, including thrombocyte and leukocyte count, liver enzymes (ALT, AST), and spleen size measurements, were analyzed. Figure 1 illustrates the changes before surgery vs. the last follow-up. A clinically significant increase in platelet count was observed in five out of six patients long term, increasing from a mean of 63 × 10^9^/L preoperatively to 154 × 10^9^/L at the final follow-up. The first patient had persistent thrombocytopenia, and the third patient showed a smaller increase in platelet count compared to the others. Additionally, leukocyte count increased markedly in five out of six patients, with persistent leukocytopenia noted in the first patient. Changes in liver enzymes (ALT, AST) were less pronounced; slight elevations were noted in patients 1, 2, and 3. All five patients who demonstrated improved blood counts after surgery also had a corresponding decrease in spleen size.

## 4. Discussion

Portal pressure exceeding 10 mmHg (or a hepatic venous pressure gradient > 4 mmHg) defines portal hypertension (PH). When portal pressure reaches 12 mmHg or higher, complications such as splenomegaly, cytopenias, or variceal bleeding may develop [9,14]. The predominant cause of PH in children is extrahepatic portal venous obstruction (EHPVO), accounting for ~70% of cases. Nevertheless, PPH is a rare disease, occurring in only ~1 in 100,000 children [6,15,16]. Major risk factors for developing EHPVO in childhood include neonatal umbilical vein catheterization, transfusions, neonatal sepsis, abdominal infections, and congenital cardiovascular malformations, whereas in adults, the leading cause of portal vein thrombosis is hypercoagulability [16,17]. In our series, three (50%) patients had identifiable risk factors in their history, such as neonatal sepsis, bacterial infection, or cardiovascular malformation.

A concerning aspect is that children with portal hypertension often exhibit no symptoms until complications arise, which then require urgent intervention [18,19]. In our study, two (33%) patients were incidentally found to have an enlarged spleen and thrombocytopenia, indicating hypersplenism and possibly portal hypertension. The remaining four (67%) patients presented with symptomatic portal hypertension: three of these children (50% of the total) had advanced hypersplenism symptoms (left upper quadrant pain, easy bruising, frequent infections), among whom one also had epistaxis with large esophageal varices requiring urgent sclerotherapy. The fourth patient (17%) initially presented with an acute variceal hemorrhage as the first manifestation of portal hypertension, necessitating emergency endoscopic intervention.

Pharmacological therapy for portal hypertension with nonselective β-blockers (e.g., propranolol, nadolol) aims to decrease portal pressure and prevent variceal bleeding by reducing cardiac output and inducing splanchnic vasoconstriction through blockade of β-1 and β-2 receptors [20,21]. While these medications are well established in adults, pediatric data are scarce (mostly from limited case series), so they should be used with caution in children [22]. Conversely, endoscopic interventions such as sclerotherapy and variceal band ligation are commonly employed in children and effectively manage complications like variceal bleeding [20,23]. However, these measures do not alter the disease’s natural course and necessitate repetitive procedures [16]. At our tertiary pediatric centre, approximately four new cases of prehepatic portal hypertension are diagnosed each year. The majority of these patients are managed conservatively using endoscopic and medical therapy. Endoscopic interventions, including variceal band ligation or sclerotherapy, are routinely performed to control or prevent variceal bleeding. Medical treatment with non-selective β-blockers (such as propranolol or carvedilol) is often added to reduce portal pressure. Surgical shunting is reserved for selected patients who continue to show significant hypersplenism or variceal bleeding despite optimal conservative management. The six patients described in our series represent all surgically treated children with prehepatic portal hypertension at our institution during the study period, whereas all other cases were successfully managed with medical and endoscopic approaches alone.

The surgical management of PPH evolved over decades, from non-selective portosystemic shunts (e.g., mesocaval, portocaval) to selective mesoportal shunts. All shunt procedures relieve variceal pressure to control bleeding, but only mesoportal shunting (e.g., MRS) restores hepatopetal flow and can reverse the long-term consequences of reduced liver perfusion [10]. Non-selective shunts such as the mesocaval and portocaval divert most portal blood directly to the systemic circulation, connecting the superior mesenteric vein or the portal vein to the inferior vena cava. In contrast, the splenorenal shunt selectively connects the splenic vein to the left renal vein, decompressing varices while partially preserving portal flow to the liver. The most recent mesoportal technique, the Meso-Rex shunt, directs flow from the superior mesenteric vein to the intrahepatic left portal vein using a venous conduit. MRS is considered superior to non-selective shunts in long-term outcomes, as it reduces hypersplenism, improves mild liver dysfunction, and supports normal growth [9,24,25]. On the other hand, two systematic reviews comparing MRS and other shunts (PSS) found no clear advantage of one over the other, and one report suggested MRS may have a higher risk of shunt thrombosis [26,27]. In our series, one out of five patients treated with Meso-Rex bypass experienced a post-operative shunt thrombosis. The patient with the mesorenal shunt did not develop this complication, and his long-term outcomes were comparable to those of the Meso-Rex patients. However, given that we had only one non-Meso-Rex case and a limited follow-up duration, no definitive comparison can be made. In our long-term follow-up, we observed a marked reduction or complete eradication of esophageal varices in all cases, with no episodes of gastrointestinal bleeding after surgery. Moreover, patients demonstrated improvements in thrombocytopenia and leukocytopenia, indicating amelioration of hypersplenism.

**Figure 2:**
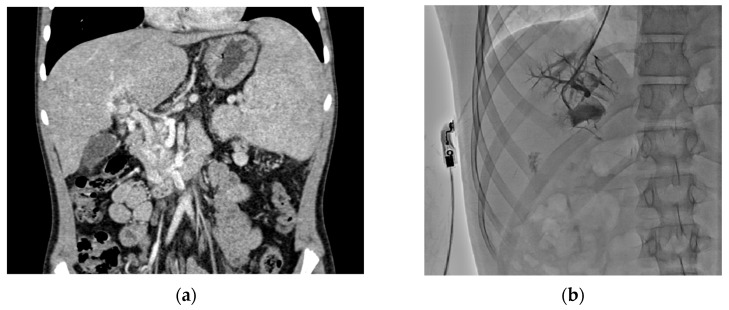
Preoperative imaging: CTA image (**a**) visualizing cavernous transformation of the portal vein with collateral vessels and retrograde portography (**b**) obtained via jugular vein catheterization and visualizing patent intrahepatic portal veins.

Preoperative imaging is crucial for planning surgery, as it provides anatomical information on vessel patency (e.g., superior mesenteric vein and left portal vein). CTA and MRA are effective non-invasive modalities to evaluate vessels in children. Additionally, hepatic vein portography can directly assess the Rex recess patency, though it is invasive and requires general anesthesia [28,29,30]. Notably, interpreting these imaging studies can be challenging, and failure to visualize a patent Rex vein does not necessarily exclude its presence [31]. In our study, all patients underwent CTA before surgery, and four patients also had portography performed (Figure 2).

Traditionally, patients with chronic liver disease are thought to have had a coagulopathy with an increased risk of bleeding as a consequence of reduced synthesis of clotting factors. More recent evidence suggests that such patients are actually in a dynamic state of ‘rebalance’ due to simultaneously altered synthesis of both procoagulant and anticoagulant factors [32,33]. This new hemostatic balance, however, appears much more fragile than the hemostatic balance in individuals with normal liver function, and patients with liver disease can readily experience both hemostasis-related bleeding and thrombotic events [33]. 

The emerging treatment paradigm for prehepatic portal hypertension is to perform Meso-Rex bypass for primary and secondary prophylaxis of variceal complications if the child has no intrinsic liver disease, a left portal vein > 2 mm, and patent mesenteric and jugular veins [13,34,35]. When performed early by experienced surgeons, this procedure can yield excellent results, significantly reducing PPH-related complications, lowering the need for endoscopic interventions, and improving survival [3,8,9,11]. Our study supports early surgery for PPH, demonstrating durable shunt function over the long term and a reduction in portal hypertension complications. We observed that patients who underwent surgery earlier in the disease course tend to have better long-term outcomes, although our sample is very small.

Consistent with prior studies, our study has several limitations. Aside from its retrospective, uncontrolled design, most patients in our series underwent surgery relatively late in the course of PPH. The small sample size—reflecting the low incidence rate in our region—limits generalization of our results and underscores the need for larger prospective studies. We also did not assess plasma ammonia levels or neurocognitive function, which would have required long-term specialized testing. Including these measures in future research could provide a more comprehensive understanding of the effects of restoring physiological portal circulation after MRS.

## 5. Conclusions

Surgical interventions, particularly Meso-Rex shunt, have emerged as effective treatments for pediatric prehepatic portal hypertension, offering long-term benefits such as reduced hypersplenism and prevention of variceal bleeding. Our experience supports early surgical intervention for improved long-term outcomes and reduced complications associated with portal hypertension. Given the long-term success of shunt procedures (particularly MRS), early consideration of surgery and diligent post-operative monitoring are crucial in managing portal hypertension in children.

## Figures and Tables

**Table 1:** Demographic and medical data.

Patient no	Age at the Time of Surgery (Years)	Gender	Risk Factors	Presenting Symptoms	Surgical Procedure	Time from PPH Diagnosis to Surgery (Years)
1	16	male	none	incidentally found splenomegaly, thrombocytopenia	Meso-Rex shunt (using femoral vein)	9
2	4	female	streptococcal sepsis at 3 weeks of age	hypersplenism	Mesorenal shunt	2
3	8	male	meconium aspiration, intubation	hypersplenism, epistaxis, esophageal varices (F2-3)	Meso-Rex shunt	6
4	12	female	none	incidentally found splenomegaly, thrombocytopenia	Meso-Rex shunt (using gastric vein)	3
5	9	female	KRT1 gene associated vascular malformation	hypersplenism	Meso-Rex shunt	2
6	6	male	none	variceal bleeding (esophageal)	Meso-Rex shunt	2

**Table 2:** Change in esophageal varices.

Patient (Shunt Type)	Before Surgery	6 Months After Surgery	4 Years After Surgery	5 Years After Surgery
1 (Meso-Rex)	F2	F1	F1	F1
2 (mesorenal)	F2	F1	F1	-
3 (Meso-Rex)	F1	F0-1	F0-1	None
4 (Meso-Rex)	F1	None	None	F0-1
5 (Meso-Rex)	F0-1	None	None	-
6 (Meso-Rex)	F2	None	-	-

**Table 3:** Postoperative shunt flow velocity measured using Doppler ultrasonography (cm/s).

Patient (Shunt Type)	6 Months After Surgery	1 Year After Surgery	3 Years After Surgery	4 Years After Surgery	5 Years After Surgery
1 (Meso-Rex)	30	-	-	13.5	-
2 (mesorenal)	19	22	27	-	22
3 (Meso-Rex)	21	38	23	24	23
4 (Meso-Rex)	-	45	-	33	25
5 (Meso-Rex)	-	68	76	33	-
6 (Meso-Rex)	45	-	-	-	-

**Table 4:** Shunt functioning.

Patient (Shunt Type)	Year of Shunt	Status at the Last Follow-Up	Shunt Flow Velocity (cm/s)
1 (Meso-Rex)	2019	Functioning poorly (4 years)	13.5
2 (mesorenal)	2018	Functioning (5 years)	22
3 (Meso-Rex)	2018	Functioning (5 years)	23
4 (Meso-Rex)	2018	Functioning (5 years)	25
5 (Meso-Rex)	2019	Functioning (4 years)	33
6 (Meso-Rex)	2024	Functioning (6 months)	45

## Data Availability

The raw data supporting the conclusions of this article will be made available by the authors on request.
